# Multilevel analysis of individual, household, and community factors influencing child growth in Nepal

**DOI:** 10.1186/s12887-019-1469-8

**Published:** 2019-04-05

**Authors:** Tim Smith, Gerald Shively

**Affiliations:** Department of Agricultural Economics, Purdue University, West Lafayette, IN 47907 USA

## Abstract

**Background:**

Childhood malnutrition and growth faltering is a serious concern in Nepal. Studies of child growth typically focus on child and mother characteristics as key factors, largely because Demographic and Health Surveys (DHS) collect data at these levels. To control for and measure the importance of higher-level factors this study supplements 2006 and 2011 DHS data for Nepal with data from coincident rounds of the Nepal Living Standards Surveys (NLSS). NLSS information is summarized at the district level and matched to children using district identifiers available in the DHS.

**Methods:**

The sample consists of 7533 children aged 0 to 59 months with complete anthropometric measurements from the 2006 and 2011 NDHS. These growth metrics, specifically height-for-age and weight-for-height, are used in multilevel regression models, with different group designations as upper-level denominations and different observed characteristics as upper-level predictors.

**Results:**

Characteristics of children and households explain most of the variance in height-for-age and weight-for-height, with statistically significant but relatively smaller overall contributions from community-level factors. Approximately 6% of total variance and 22% of explained variance in height-for-age z-scores occurs between districts. For weight-for-height, approximately 5% of total variance, and 35% of explained variance occurs between districts.

**Conclusions:**

The most important district-level factors for explaining variance in linear growth and weight gain are the percentage of the population belonging to marginalized groups and the distance to the nearest hospital. Traditional determinants of child growth maintain their statistical power in the hierarchical models, underscoring their overall importance for policy attention.

**Electronic supplementary material:**

The online version of this article (10.1186/s12887-019-1469-8) contains supplementary material, which is available to authorized users.

## Background

Human capital is a key determinant of economic growth and development [1]. Persistent malnutrition throughout early childhood can severely hinder a child’s physical and cognitive development [2] and, therefore, her accumulation of human capital. Malnutrition also increases the risk of contracting various illnesses and can deepen a child’s level of malnutrition in a highly deleterious disease-hunger feedback loop, thereby perpetuating intergenerational poverty [3, 4]. Where malnutrition is widespread, it can undermine a country’s economic performance and prospects for economic and social development. As a result, finding ways to reduce childhood malnutrition at scale remains a development imperative. A related policy-relevant question is whether policy makers and development agencies should focus interventions and investments on individuals, households, or communities, and in what proportions. Answering these questions is particularly important in the context of Sustainable Development Goals (SDG’s) two and three, which commit the international community to ending hunger and achieving health and wellbeing for people at all ages.

This paper provides empirical insights into these issues for Nepal, one of the least well-nourished countries in the world, and one where human development is frustrated by a range of economic, geographic and social challenges. A large proportion of children below five years of age in Nepal suffer from malnutrition, as indicated by population-level anthropometric indicators such as height-for-age and weight-for-age. Although the incidences of stunting and underweight fell substantially between 2001 and 2011, 41% of children under five were stunted in 2011, 29% were underweight, and 11% were acutely wasted [5]. In 2017, the Nepal Ministry of Health reported that the stunting rate continued to decline after 2011, but as of 2016, 36% of children in Nepal were stunted (HAZ < − 2.0) and 12% were severely stunted (HAZ < − 3.0) [6]. The problem of child malnutrition therefore remains pressing in Nepal, and requires analysis that rigorously asks what factors matter for the patterns observed, and at what levels. Recent reviews of maternal and child nutrition [2, 7] highlight a range of individual- and household-level factors that can influence a child’s health, nutrition and physical growth, among them mother’s health and education, access to clean water and sanitation, and food consumption and diet diversity. In Nepal, observed reductions in undernutrition over time have been traced to asset accumulation, health and nutrition interventions, gains in maternal education, and improvements in sanitation [8]. However, gaps remain in our understanding of how community factors might contribute to outcomes. These factors may be potentially important for understanding whole-population shifts in growth faltering [9, 10]. For example, as in many countries where infrastructure is weak and households are isolated, in Nepal supra-household environmental conditions such as rainfall are correlated with outcomes, along with community-level factors such as roads and markets [11, 12].

In this paper we study a range of individual, household, and community factors in relation to height-for-age and weight-for-height. We build on a conceptual framework developed by UNICEF [13] and extended by Smith and Haddad [14], who posit three distinct categories of nutritional determinants, arranged hierarchically: (i) *immediate determinants*, occurring at the child level and proximately determining outcomes; (ii) *underlying determinants*, generally occurring at the household level and mediated through immediate determinants; and (iii) *basic determinants*, i.e. those features of communities which provide the context for underlying and immediate determinants. This hierarchy translates comfortably into a three-level mixed model regression framework, which we employ to test two general hypotheses. The first is that community-level factors (specifically local food supply, the local health environment, and cultural characteristics) are relevant to explaining observed patterns of growth, even when one controls for child- and household-level characteristics. Evidence regarding this hypothesis provides insights into interventions that might prove effective in promoting child health and nutrition. The second hypothesis is that omitting these higher-level characteristics from models of child growth may lead to an overestimation of the importance of individual- and household-level factors (such as acute sickness, breastfeeding practices, mother’s education and health, and household wealth) in explaining observed variance in growth metrics.

We make two contributions. The first is that we incorporate data from multiple datasets, including Demographic and Health Surveys (DHS) and Living Standards Measurement Surveys (LSMS), matching information at geographic reference points and incorporating it under minimally onerous representation assumptions. This permits us to fill an empirical gap in the literature, by including covariates representing variables potentially amenable to policy intervention at broad scales. The second contribution is to demonstrate how hierarchical modelling techniques can be used to measure the relative contribution to and importance of relationships between child-level anthropometry and household- and community-level covariates in a way that constitutes a methodological improvement over standard linear regression models. We are not the first to answer questions about childhood nutrition by considering data observed at different levels in this way, however, and have drawn on the small but focused literature on these topics. The most closely related study applies similar techniques to earlier data from the NDHS, but uses discrete measures of underweight and stunting, and is unable to include the kinds of community variables available in the NLSS. Therefore, while previous research [15] reaches similar conclusions regarding household and individual factors, we are able to incorporate and study the role of community determinants in a more complete manner. The broader literature on multilevel models of childhood nutrition outcomes [16–20] also provides guidance regarding selection of variables and the interpretation of results, but these papers either focus on allowing household parameters to vary over space or on including hierarchical random effects, rather than integrating community characteristics through the hierarchical structure as we do.

## Methods

### Data sources

To estimate our models we stack data from two child-level datasets constructed from the 2006 and 2011 Nepal Demographic and Health Surveys (DHS). We then merge to these data information from the 2004 and 2010 Nepal Living Standards Surveys (NLSS). The DHS surveys include our dependent variables for children under five years of age, as well as child, mother and household characteristics that have been shown in past studies to be relevant to explaining child growth. The NLSS includes measures of agricultural activity, access to services, infrastructure, and incomes at the individual and household levels. The NLSS did not visit the same households as the DHS, so we cannot directly match household information. However, both surveys used the same district definitions and identification codes. This allows us to aggregate household observations from the NLSS up to the district level, and then match a set of district-level NLSS variables to DHS households based on district and year combinations. To our knowledge, there is no publicly available crosswalk that would allow a researcher to match children across surveys or to match geographic data at a finer scale (e.g. subdistrict, village, or municipality). Therefore, we do not attempt to produce any matches at scales finer than the district. We match 2004 NLSS data to the 2006 DHS, and 2010 NLSS data to the 2011 DHS. The 2006 DHS includes 5237 children, and the 2011 DHS includes 2335 children. When combined, these datasets provide anthropometric information on 7572 children under age five. A total of 39 children were omitted due to missing values for independent variables, leaving 7533 child-level records for analysis. The validity of our DHS-NLSS matching rests on the assumption that these measures of community characteristics from the NLSS are reliable measures of the more general circumstances surrounding a child subsequently observed in the DHS. To account for differences in lag lengths and potential observed and unobserved heterogeneity in trends across time and space we use survey year and birth year controls. Use of these data did not require institutional review because respondents previously provided informed consent and were rendered anonymous before the data were released to us for analysis.

Our dependent variables are the child’s height-for-age z-score (HAZ) and weight-for-height z-score (WHZ). Z-scores measure the dispersion of the indicator as standard deviations around a reference population median, and are calculated as:

1 $$ {z}_i=\frac{x_i-\overline{x}}{\sigma_x} $$

where x_*i*_ is the individual observation and x̅ and σ_*x*_ are the median and the standard deviation of the reference population. Z-scores were calculated using the WHO’s current Child Growth Standards reference population [21]. Our use of continuous z-score outcomes is noteworthy because many studies use a binary dependent variable to indicate stunting (HAZ < − 2.0) or wasting (WHZ < − 2.0) [15, 16, 22, 23]. Z-score cutoffs (e.g. -2.0 for stunting and wasting or − 3.0 for severe stunting or severe wasting) can mask important information about the entire distribution of outcomes and their use discards information about that distribution, a fact recognized at the time z-scores were introduced by the WHO [24]. Elsewhere [25–27] it has been argued that the widely-accepted − 2.0 cutoff is arbitrary, with little biological basis for a threshold. Using a continuous measure in place of a binary indicator allows us to capture the intensity of growth faltering in the population. Z-scores used in this analysis are distributed normally, although plots of z-scores against quantiles of the normal distribution do reveal slight departures from normality in the extreme tails of the distributions, but not to a degree that is detrimental to the analysis or amenable to correction via a monotonic transformation of the data.

Among *immediate determinants*, we include a large set of child-level variables that have been shown to be correlated with child growth in Nepal and elsewhere. These include the child’s age (in months), sex, and twin status, as well as two indicators of acute disease symptoms (diarrhea in the two weeks prior to anthropometric measurement and fever in the same period) as these are known to place demands on a body’s physical resources [16]. Given the importance of breastfeeding patterns in determining nutrition, health and physical growth [2, 15], we include a binary variable indicating whether a child was being breastfed at the time of measurement, along with the total number of months of breastfeeding. In further recognition of the importance of a mother’s status and education [17, 27–33], as well as natal and perinatal health in early childhood development [2, 34–37], we also include a set of maternal characteristics that are tied to children. These include a woman’s body mass index (BMI), her age at birth (in years), her education (in years), and a binary indicator of her hand-washing opportunities (coded as one if a place for handwashing with running water was available in the household, and zero otherwise).

We also include the squares of child’s age and breastfeeding duration to allow for the possibility that the relationship between HAZ and these time variables is nonlinear. This could be the case if, for example, households are, on average, better at providing nutrition for younger and older children compared to children in the middle range of ages in our sample, or if breastfeeding after a certain age is a less effective way of delivering nutrition. Including the squares of these terms allows the marginal effect of the variable in question to depend on the value of that variable as well as the estimated coefficients, so that if the relationship between HAZ and the variable changes across the variable’s range, we can detect that difference when we fit the regression.

At the household level, we account for several *underlying determinants*. One is membership in the Dalit caste. While the caste system was officially abolished in Nepal 1962, evidence suggests continued discrimination, which may affect a child’s status in ways not captured by the other variables included at this level [38]. We control for economic status via a wealth index, measured as the household’s quintile value on an index of wealth generated by DHS analysts applying weights to observed household assets using principal components analysis. Elsewhere, this has been used as a measure of household socioeconomic status [17, 18, 33]. A substantial body of research suggests that economic wellbeing has a positive effect on children’s nutritional status and growth [15, 31, 32, 39]. We also include indicators of water and fuel sources, the former in recognition of the importance of waterborne diseases to nutrition and health [40, 41], and the latter in recognition of the potential importance of indoor air quality for upper respiratory health and child growth [42, 43]. Indoor air pollution from tobacco smoke and the burning of biomass fuels is common in Nepal and have health effects with implications for child growth [44, 45]. We therefore include an indicator for the type of fuel used (one if the household used biomass too cook; zero otherwise). We also include altitude (in meters above sea level) as a control variable. We expect altitude to control for multiple factors that could impact growth. Altitude and linear growth are likely to be negatively correlated due to remoteness, and also because the reduced oxygen content of air at altitude may impair growth [46, 47].

We also incorporate community-level *basic determinants*. Previous multilevel regression work on child mortality and stunting included distance to the nearest health facility, community-level rates of education attainment, and infrastructure [29]. Our expectation is that omitting higher-level factors could lead to mistaken inference regarding point estimates on child- and household-level variables, and mask the importance of non-nutrition interventions of interest to policy makers. Recent work from Nepal, for example, demonstrates the importance of food markets in mitigating the effects of climate on linear growth [10], and the role of transportation infrastructure in moderating food prices [48] and explaining patterns of child growth [49, 50].

All district-level variables are derived from either the NLSS or from Nepal census data. Because child and household-level food consumption variables are not available in the DHS, we measure the percentage of NLSS respondents who reported their food consumption within the last month as inadequate. Food shortages are determined at least partially by factors which affect all households in a district, such as weather, soil characteristics, and food prices. We also include a measure of market access (a commercialization ratio computed as the proportion of NLSS households in a district that reported selling some amount of their agricultural output). We include an indicator of access to healthcare (the median reported distance to the nearest hospital, in minutes on foot) and a measure of community-level hygiene (the percentage of Village Development Committees (VDCs) in a district that were declared open defecation free at the time of the survey). Finally, to control for overall social conditions, we include an ethnicity indicator (the percentage of a district’s population that belongs to a marginalized ethnic or caste group, calculated from census data), and a measure of gender equity (calculated from census data as the ratio of female students to total students in a district). Descriptive statistics for all variables are presented in Table 1. These statistics are included primarily for reference, but some summaries merit particular attention. First, we note the quite low average HAZ values, with a mean of − 1.88, implying that the average child is very close to the stunting cutoff, a fact that underscores the urgency of understanding undernutrition in this context. Average levels of maternal education are also extremely low, which is concerning given the importance of this variable in the literature. It is, however, worth noting that the average child is breastfed for about a year, approximately consistent with WHO guidelines, a positive outcome for this particular period in children’s lives.

**Table 1:** Descriptive statistics for all variables used in the regressions

Variable	Units	Mean	Std. Dev.	Min.	Max.
Child level (*n* = 7572)					
HAZ	Standard deviations	−1.88	1.35	−5.96	4.59
WHZ	Standard deviations	−0.79	1.08	−4.94	4.07
Age	Months	30.0	17.1	0	59
Age^2^	Months^2^	1193	1054	0	3481
Twin status	0/1 indicator	0.01	0.10	0	1
Female	0/1 indicator	0.49	0.5	0	1
Breastfeeding	0/1 indicator	59.3%	49.1%	0	1
Breastfeeding duration	Months	12.1	14.5	0	59
Breastfeeding duration^2^	Months^2^	356	605	0	3481
Fever in past two weeks	0/1 indicator	0.19	0.39	0	1
Diarrhea in past two weeks	0/1 indicator	0.13	0.34	0	1
Mother’s education	Years	2.8	3.8	0	14
Hand washing access	0/1 indicator	0.62	0.48	0	1
Mother’s BMI	BMI value	20.6	2.7	14.0	36.9
Mother’s age at birth	Years	24.9	5.9	13	47
Household level (*n* = 5450)					
Wealth	Quintile (1–5)	2.7	1.4	1	5
Water purification	0/1 indicator	0.13	0.34	0	1
Altitude	Meters	836	730	46	3189
Ethnicity (Dalit)	0/1 indicator	0.163	0.37	0	1
Biomass fuel use	0/1 indicator	0.87	0.34	0	1
District level (*n* = 75)					
Food short	% of households	26.6%	18.7%	0.0%	91.7%
Educational Equity	% girls in schools	48.7%	3.3%	37.7%	53.8%
Marginal	% of households	47.0%	19.1%	6.1%	85.7%
Commercial sales	% of households	45.1%	20.9%	0.0%	91.7%
Hospital distance	minutes by foot	403	642	5	3600
Open defecation prevalence	% VDC’s ODF free	11.1%	21.9%	0%	100%

Merging data from different surveys conducted over different time frames, as we do here, is not ideal, but given the limited availability of data, and the fact that the DHS does not include the data we need to relate child growth to local the social and economic conditions we emphasize, it is necessary. Certain factors mitigate concerns about this approach, however. First, we note that districts in Nepal are quite small compared to the top-level subnational administrative units in other countries; as of the 2011 census, the most populous district by far was Kathmandu, with around 1.7 million residents, a population scale more comparable to Indian districts or U.S. counties than to states in either country. At this scale, we are confident that measures of the local conditions we emphasize are relevant for children’s nutritional outcomes, and while we would prefer to use data at the village or municipality level, the data necessary to do this are, to our knowledge, either nonexistent or inaccessible. In a nationally representative survey like the NLSS, we expect sample means and medians at the district level to act as reasonably good estimators of the population analogs, and we restrict our analysis to measures of central tendencies of variables, which should reflect general social and economic conditions. We therefore expect that, while our approach may introduce noise, it is unlikely to introduce bias. To test this conjecture, we conducted Kolmogorov-Smirnov tests comparing residuals from regressions which include only variables derived from the DHS to residuals from regressions which include the district data. If the non-DHS variables were systematically correlated with the residuals, we would see differences between these distributions. We fail to reject the null hypothesis of no difference in all cases at the 95% confidence level, however.

### Empirical strategy

Using multilevel models for z-scores has conceptual and technical advantages. When the level of observation at which the dependent variable occurs is nested within other levels—for example children nested in households and districts—including higher-level characteristics as child-level predictors can lead to the misstatement (generally understatement) of standard errors, as one value will be replicated across all members of the same group. With a multilevel model, the value is applied once, at the group level, and information from the pooled regression can help generate reliable estimates even for groups with very low numbers of first-level observations [51]. Using multilevel models also allows us to include error terms at each level, which makes it possible to track changes in variance at each level across models. Taken together, these properties give multilevel models a substantial advantage over classical regression models when dealing with hierarchically structured data, like those analysed here [15].

The specific form of our multilevel regression models is given by eqs. (2, 3, and 4):

2 $$ {Z}_i={\alpha}_{jk}+\beta {X}_i+{e}_i\kern1.25em i=1,\dots, I $$

3 $$ {\alpha}_{jk}={\gamma}_0^j+{\gamma}_k+{e}_j\kern0.1em \mathrm{for}\kern0.1em j=1,\dots, J,k=1,\dots, K $$

4 $$ {\gamma}_k={\lambda}_0^k+{\lambda}_k{D}_k+{e}_k\kern0.1em \mathrm{for}\kern0.2em k=1,\dots, K $$

where *Z*_*i*_ is the z-score for child *i* in household *j* in district *k*, *α*_*jk*_ and *β* are intercept and coefficient vectors for individual-level variables *X*_*i*_, ${\gamma}_0^j$ is a household-specific intercept, and *γ*_*k*_ are district-level intercepts, each of which is a function of district-level variables *D*_*k*_,district-level coefficients *λ*_*k*_, and the district-level intercepts ${\lambda}_0^k$. Finally, *e*_*i*_, *e*_*j*_, and *e*_*k*_ are error terms at each level. In this specification, *α*_*jk*_ does not vary in household characteristics, but including a household level allows us to estimate household intercept terms and variance components. The expanded variance terms allow us to account for variance arising at child, household and district levels. We model a child’s z-score as a function of variables specific to the child (including characteristics of the mother and household). We model variance at the district level as a function of district-level variables. We account for household-level variance, but given the low ratio of children under age five to households, the dataset does not support inclusion of separate household-level covariates at the household level.

## Results

The main regression results are presented in Table 2 (for HAZ) and Table 3 (for WHZ). Models are organized as follows. Model 1 is a null model, in which no predictors are included but the variance is partitioned into between-child and between-district components by adding district-level shifts in the child-level random intercept value. Model 2 adds predictors at the child level, while maintaining district random intercepts. Models 3 and 4 add different sets of predictors at the district level. For HAZ only, Model 5 includes a district-level sanitation variable. In all cases, continuous variables included as explanatory variables have been standardized, so that the coefficient for any continuous variable is interpreted as the estimated change in the z-score resulting from a one standard deviation change in that variable. The exception to this standardization is the wealth index variable which is centered on its median value of three.

**Table 2:** Regression results for three-level (child-household-district) models of HAZ

	Model 1	Model 2	Model 3	Model 4	Model 5
Age (months)	–	−0.235*** (0.0583)	−0.233*** (0.0583)	−0.234*** (0.0583)	−0.234*** (0.0582)
Age^2^ (months squared)	–	0.177*** (0.0405)	0.177*** (0.0405)	0.178*** (0.0404)	0.178*** (0.0404)
Female (0/1)	–	0.00313 (0.0260)	0.00476 (0.0260)	0.00487 (0.0260)	0.00463 (0.0260)
Twin (0/1)	–	−0.651*** (0.144)	−0.648*** (0.144)	− 0.643*** (0.144)	−0.644*** (0.143)
Still breastfeeding (0/1)	–	0.765*** (0.228)	0.768*** (0.228)	0.768*** (0.228)	0.766*** (0.228)
Months breastfeeding (months)	–	−0.482*** (0.121)	−0.482*** (0.121)	− 0.481*** (0.121)	−0.480*** (0.121)
Months breastfeeding^2^ (months squared)	–	0.126*** (0.0338)	0.126*** (0.0338)	0.126*** (0.0337)	0.125*** (0.0337)
Fever in last two weeks (indicator)	–	0.0245 (0.0350)	0.0234 (0.0350)	0.0240 (0.0350)	0.0239 (0.0350)
Diarrhea in last two weeks (indicator)	–	−0.0743 (0.0405)	−0.0743 (0.0405)	− 0.0742 (0.0405)	− 0.0737 (0.0405)
Mother’s education (years)	–	0.159*** (0.0186)	0.161*** (0.0186)	0.161*** (0.0186)	0.161*** (0.0186)
Access to handwashing (indicator)	–	0.166*** (0.0339)	0.160*** (0.0340)	0.164*** (0.0339)	0.163*** (0.0339)
Mother’s BMI (BMI units)	–	0.113*** (0.0153)	0.113*** (0.0153)	0.113*** (0.0153)	0.113*** (0.0153)
Mother’s age at birth (years)	–	0.00285 (0.0147)	0.00270 (0.0147)	0.00250 (0.0147)	0.00240 (0.0147)
Wealth Index (quintile, 1–5, centered)	–	0.0870*** (0.0151)	0.0854*** (0.0152)	0.0888*** (0.0151)	0.0884*** (0.0151)
Water purification (0/1)	–	0.0603 (0.0515)	0.0558 (0.0514)	0.0542 (0.0512)	0.0569 (0.0512)
Year (1 = 2011, 0 = 2006)	–	0.129*** (0.0329)	0.135*** (0.0372)	0.125*** (0.0378)	0.124** (0.0377)
Altitude (m.a.s.l.)	–	−0.170*** (0.0267)	−0.182*** (0.0282)	− 0.174*** (0.0261)	− 0.179*** (0.0261)
Mother is a Dalit (0/1)	–	− 0.124** (0.0409)	− 0.132** (0.0410)	−0.122** (0.0409)	− 0.121** (0.0409)
Biomass usage (0/1)	–	−0.0852 (0.0593)	− 0.0882 (0.0594)	− 0.0752 (0.0595)	− 0.0798 (0.0596)
Constant	−1.913*** (0.0427)	−2.609*** (0.151)	−2.601*** (0.151)	−2.614*** (0.151)	−2.600*** (0.151)
Birth timing controls	Month	Month	Month	Month	Month
Residual variance	1.367*** (0.0381)	0.935*** (0.0269)	0.935*** (0.0270)	0.935*** (0.0270)	0.935*** (0.0270)
District variance	0.109*** (0.0222)	0.0333*** (0.0085)	0.0277** (0.0086)	0.000352 (0.00573)	1.71E-15 (8.61e-15)
Household variance	0.351*** (0.0360)	0.395*** (0.0282)	0.388*** (0.0281)	0.388*** (0.0281)	0.388*** (0.0281)
Food shortage† (% reporting shortage)	–	–	0.00568*** (0.00490)	–	–
Gender equity† (female enrollment ratio)	–	–	0.0091*** (0.0059)	0.0043*** (0.0054)	0.0049*** (0.0055)
Marginal† (% marginalized)	–	–	–	0.0244*** (0.0114)	0.0237*** (0.0100)
Commercial† (% selling food)	–	–	–	0.0034 (0.0075)	0.00194 (0.0084)
Hospital distance† (minutes on foot)	–	–	–	0.0121*** (0.0092)	0.0123*** (0.0094)
ODF free† (% of VDCs)	–	–	–	–	0.00283*** (0.0038)
Observations	7533	7533	7533	7533	7533
Total Variance	1.827	1.3633	1.3507	1.3234	1.323
Level 1 R-squared	–	0.32	0.32	0.32	0.32
Level 3 R-squared	–	0.69	0.75	0.99	0.99
Overall R-squared	–	0.25	0.26	0.28	0.28
District ICC	0.0597	0.0244	0.0205	0.0003	0.0000
Household ICC	0.252	0.3144	0.3078	0.2937	0.2932
Log-Likelihood	−12,743.5	−11,696.3	−11,690.4	−11,686.2	−11,685.4
AIC	25,494.95	23,460.55	23,452.85	23,448.44	23,448.73

Notes: Standard errors presented in parentheses. † indicates variable has been standardized

Use of these data did not require institutional review because respondents previously provided informed consent and were rendered anonymous before the data were released to us for analysis

**Denotes statistical significance at the 5% confidence level

***Denotes statistical significance at the 1% confidence level

**Table 3:** Regression results for three-level (child-household-district) models of WHZ

	Model 1	Model 2	Model 3	Model 4
Age (months)	–	0.0920 (0.0517)	0.0921 (0.0517)	0.0944 (0.0517)
Age^2^ (months squared)	–	−0.107** (0.0359)	−0.107** (0.0359)	−0.109** (0.0359)
Female (0/1)	–	0.00719 (0.0230)	0.00744 (0.0229)	0.00635 (0.0229)
Twin (0/1)	–	−0.461*** (0.125)	−0.464*** (0.124)	− 0.465*** (0.124)
Still breastfeeding (0/1)	–	0.325 (0.202)	0.327 (0.202)	0.334 (0.202)
Months breastfeeding (months)	–	−0.374*** (0.107)	−0.376*** (0.107)	− 0.380*** (0.107)
Months breastfeeding^2^ (months squared)	–	0.159*** (0.0299)	0.159*** (0.0299)	0.161*** (0.0299)
Fever in last two weeks (indicator)	–	−0.135*** (0.0307)	−0.135*** (0.0307)	− 0.138*** (0.0307)
Diarrhea in last two weeks (indicator)	–	−0.126*** (0.0357)	− 0.125*** (0.0357)	−0.124*** (0.0356)
Mother’s education (years)	–	0.0371* (0.0161)	0.0359* (0.0161)	0.0377* (0.0160)
Access to handwashing (indicator)	–	0.0287 (0.0291)	0.0271 (0.0292)	0.0295 (0.0292)
Mother’s BMI (BMI units)	–	0.236*** (0.0133)	0.235*** (0.0133)	0.235*** (0.0132)
Mother’s age at birth (years)	–	−0.0229 (0.0128)	−0.0228 (0.0128)	− 0.0214 (0.0128)
Wealth Index (quintile, 1–5, centered)	–	0.0254* (0.0129)	0.0257* (0.0130)	0.0240 (0.0129)
Water purification (0/1)	–	0.0858 (0.0443)	0.0845 (0.0443)	0.0804 (0.0441)
Year (1 = 2011, 0 = 2006)	–	0.0973*** (0.0283)	0.0893** (0.0301)	0.105*** (0.0317)
Altitude (m.a.s.l.)	–	0.136*** (0.0208)	0.137*** (0.0217)	0.131*** (0.0208)
Mother is a Dalit (0/1)	–	−0.0376 (0.0350)	−0.0372 (0.0351)	−0.0337 (0.0350)
Biomass usage (0/1)	–	0.0554 (0.508)	0.0582 (0.0509)	0.0711 (0.0511)
Constant	−0.739*** (0.0316)	−1.028*** (0.133)	−1.023*** (0.133)	−1.040*** (0.133)
Birth timing controls	Month	Month	Month	Month
Residual variance	0.85*** (0.0244)	0.781*** (0.0225)	0.78*** (0.0225)	0.778*** (0.0224)
District variance	0.0574*** (0.0120)	0.0149** (0.00462)	0.0138** (0.00481)	0.00151 (0.00461)
Household variance	0.263*** (0.0240)	0.233*** (0.0221)	0.231*** (0.0221)	0.23*** (0.0220)
Food shortage† (% reporting shortage)	–	–	0.00263*** (0.00351)	–
Gender equity† (female enrollment ratio)	–	–	0.00174*** (0.00251)	0.000391 (0.00222)
Marginal† (% marginalized)	–	–	–	0.0120*** (0.00670)
Commercial† (% selling food)	–	–	–	3.92e-12*** (2.18e-11)
Hospital distance† (minutes on foot)	–	–	–	0.0130*** (0.00668)
Observations	7533	7533	7533	7533
Total Variance	1.170	1.029	1.025	1.010
Level 1 R-squared	–	0.08	0.08	0.09
Level 3 R-squared	–	0.74	0.76	0.97
Overall R-squared		0.12	0.12	0.14
District ICC	0.049	0.015	0.013	0.001
Household ICC	0.274	0.241	0.239	0.229
Log-Likelihood	−11,085.970	−10,705.570	−10,704.630	−10,696.715
AIC	22,179.950	21,479.140	21,481.260	21,469.430

Note: Standard errors presented in parentheses. † indicates variable has been standardized. ODF variable omitted from the WHZ regression

**Denotes statistical significance at the 5% confidence level

***Denotes statistical significance at the 1% confidence level

Model 1 demonstrates that a relatively low proportion of the overall variance in anthropometric measures occurs between districts (approximately 6% for HAZ and 5% for WHZ). As the results for Model 2 shows, conventional predictors of malnutrition, occurring at the child and household level and modeled at the child level in the hierarchical regressions, are, for the most part significantly associated with HAZ and WHZ, with expected signs. Negative and statistically significant correlates for HAZ include child’s age in months (mean = 30; std. dev. = 17.1), twin status (mean = 0.01; std. dev. = 0.10), altitude in meters (mean = 836; std. dev. = 730), and minority status (mean = 0.16; std. dev. = 0.37). Results for WHZ, summarized in Table 3, are similarly intuitive. Negative and statistically significant correlates for WHZ also include indicators for acute sicknesses: fever in the past two weeks (mean = 0.19 and std. dev. = 0.39) and diarrhea in the past two weeks (mean = 0.13; std. dev. = 0.34), both of which are associated with relatively large reductions in WHZ. Positive and statistically significant correlates for HAZ and WHZ include mother’s education in years (mean = 2.8; std. dev. = 3.8), mother’s BMI (mean = 20.6; std. dev. = 2.7) and the household wealth quintile. Surprisingly, the coefficient on the water treatment indicator is not significantly different from zero at standard test levels in these models.

**Fig. 1:**
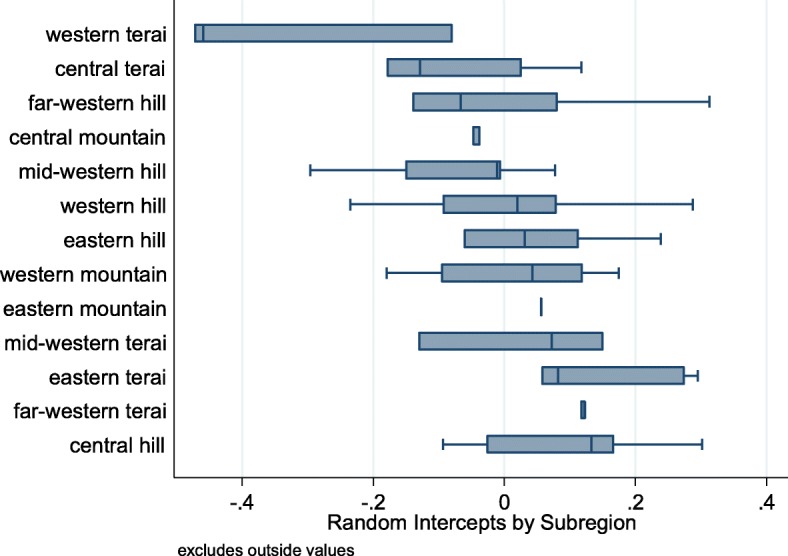
District Intercepts by Sub-region

To compare different specifications of the upper-level portions of the model, we run models for each of the three community-level factors of interest: the food supply, the health environment, and cultural factors. Comparisons across models 2–5 for HAZ (Table 2) and models 2–4 for WHZ (Table 3) indicate that point estimates for the individual- and household-level variables are similar in sign, magnitude and significance across different upper-level specifications. We compare the performance of these models using AIC and R-squared measures, computing and comparing variance from each model overall and at each level relative to the variance in Model 1. As results in Table 2 show, including district-level predictors improves the model of HAZ, compared to including only child-level predictors with district random intercepts. Adding district-level measures for food shortages or gender equity results in measurable improvements in goodness of fit. In the WHZ models (Table 3) the coefficients are smaller, but the improvements in goodness of fit have a similar magnitude, and follow similar patterns. Improvements to model fit when upper-level predictors are added to the model are confirmed by the AIC and Likelihood Ratio (LR) tests. Partitioning upper-level variance into specific factors, rather than simply leaving between-group heterogeneities controlled but completely unexplained, clarifies the model’s predictions. As an example, Model 4 partitions almost all of the district intercept variance in HAZ and WHZ into variances in specific parameters. Characteristics of children and households explain most of the variance in height-for-age and weight-for-height, with statistically significant but relatively smaller overall contributions from community-level factors. Approximately 6% of total variance and 22% of explained variance in HAZ occurs between districts. For WHZ, approximately 5% of total variance, and 35% of explained variance occurs between districts. Figure 1 further illustrates the district-level variances by showing the average district-level intercepts from Model 1 for HAZ computed at the sub-region level.

As a robustness check, Table 4 reports intraclass correlation coefficients (ICCs) under alternative upper-level specifications. Relative to using districts, using primary sampling units (PSUs) or Wards to define communities does not increase upper-level variance substantially, relative to the proportional increase in the number of groups. As a further check on robustness of the results, a series of alternative regressions are reported in (Additional file 1). These include parallel regressions that add birth year fixed effects (Tables S1 and S3) and a set of regressions that cluster standard errors at the district level (Tables S2 and S4). Signs, magnitudes and statistical significance of point estimates are broadly similar to those reported in Table 2 and Table 3. Table S5 reports variance components for all included variables, splitting variance contributions at household and district levels into between-group and within-group proportions.

**Table 4:** Intraclass correlation coefficients for alternative model specifications

Upper-Level Unit	Null Model	With Child Predictors
Household	24.4%	30.0%
PSU	10.9%	8.4%
Ward	9.8%	4.5%
District	6.1%	3.0%
Sub-region	3.1%	1.2%
Ecological Zone	1.3%	0.0%

## Discussion

Results suggest that individual- and household-level characteristics matter more than district-level factors in explaining HAZ and WHZ patterns. The relatively low proportion of between-district variance in the null model can be explained by a short list of household level variables. However, factors expected to play a role do improve fit, and many show significant variance in their effects across districts, a finding from the multilevel models which would go undetected in a classical regression model. Access to healthcare, cultural and ethnic characteristics, and aspects of the food economy explain variance that remains after the inclusion of household variables in the multilevel model. This pattern is consistent with the relevant theory. However, while these features make the models more reliable, they do not substantially improve the fit of the model or the relative importance of household characteristics. This result is consistent with past work on child growth using multilevel models, where differences in first-level parameter values were observed between Africa and Asia, but not within continents [30], and where between-community variance has been reported as low [16, 19, 20]. In studies that included community-level covariates [19, 30], such variables were found to be less influential for child growth and health than individual and household covariates.

The canonical child and household level variables included in the first level of the HAZ and WHZ models have strong and significant coefficients. This finding persists throughout the varied district-level specifications. The fit statistics for the random intercept model (Model 2) suggest that heterogeneities in levels of these variables explains the majority of between-district variance, further emphasizing their importance in explaining children’s nutrition. That these variables are strongly associated with HAZ and WHZ in a multilevel model that controls for place-based characteristics reinforces their importance, compared to an ordinary least squares regression model.

Between-district variance is fairly limited, however. The ICC of Model 1 shows that only 6% of variance in HAZ occurs between districts. This suggests the benefits of the multilevel approach, both in terms of reinforcing the role of first-level variables and in terms of modelling district characteristics, is modest. As the alternative second-level specifications in Table 4 demonstrate, using PSUs or Wards as the denomination of communities does not increase the second-level variance substantially, relative to the proportional increase in number of groups, relative to districts. Similarly, including a household-level random intercept shows that a large percentage of variation occurs between households, conditional on individual- and district-level covariates. Conventional wisdom for Nepal and other countries with geographically isolated populations suggests location may be a key determinant of malnutrition, but this perspective is not wholly supported by these results. However, we do find an altitude effect in child growth, a pattern that future work might seek to corroboration elsewhere. Although one might expect to find a strong correlation between wealth and growth outcomes, we find that maternal education is relatively more important for HAZ than household wealth: a one standard deviation change in maternal education has almost twice as strong an effect on HAZ as a one quintile shift in a household’s wealth index position. Results for WHZ confirm the short-term importance of acute sickness, mother’s BMI and breastfeeding practices for maintaining weight.

The consistency of the first-level coefficients when district random slopes are included in the models reinforces the importance of these factors to our understanding of child growth patterns, consistent with the literature we cite above. [14–19, 28–32] In general, the interpretation of higher-level parameters is less straightforward than the interpretation of coefficients on first-level variables because the model generates coefficient estimates for each district-variable combination. This provides variance parameters for each estimated upper-level effect, and for the random intercept values generated for each group unit, e.g. the district, household, or ecological zone. We note that adding additional upper-level parameters presents a computational challenge, because there are only 75 districts in Nepal. As a result, adding parameters at the district level rapidly reduces the ratio of groups to parameters, which can undermine statistical power and cause models to fail to converge. Our response is to run sub-families of models for each of the three community-level factors included in the conceptual model for which we have good data, namely the food supply, the health environment, and cultural factors. These models individually underscore the importance of these variables. Sufficient collinearity among district-level variables suggests that there is little to be gained by adding additional variables to the model. Undoubtedly, these variables play similar roles and may serve equally well as proxies for *basic determinants* in models of HAZ and WHZ.

Several individual- and household-level variables known to affect long-term growth through children’s health are not included, due to data limitations. Birth order has been identified as an important determinant of growth outcomes, due to its influence on intra-household distribution of resources and on parental care [52], but we were unable to construct a birth order variable for the 2006 DHS sample. A more detailed approach to measuring breastfeeding practices could also improve this analysis, particularly if such an approach were to include a careful measure of the exclusivity of breastfeeding. In Nepal, where breastfeeding is widespread [15], exposure to proper breastfeeding practices may be more important than simple exposure to breastfeeding [53], which is all we are able to measure and analyze.

Our results confirm positive associations between child growth and maternal education found elsewhere, and replicate findings for wealth, measured with the same index we use here [8, 54, 55]. While the coefficient on the wealth index is consistent across models, it is small. This is largely because many of the other household variables, including maternal education, are likely related to wealth, and in some cases – such as our sanitation variables – may be channels through which wealth is related to HAZ. In an unreported bivariate regression of HAZ on the wealth index, the coefficient is approximately 0.24, and significant at the 1% confidence level, but as we add maternal education, maternal BMI, handwashing access, and water purification to the model conditional on the other variables, its value drops to the level we see in Table 2, with the largest decrease coming from adding maternal education. Previous work for Nepal found no significant association between linear growth and an improved water supply, although a positive association between HAZ and access to a flush toilet was found [54, 56]. In our models, the water variable is significant in only a few regressions, and even then, only marginally so (at the 90% confidence level). Rather than suggesting that sanitation is unimportant to child growth, however, this result more likely indicates that our proxy for sanitation is poorly measured or less relevant for a long-term indicator such as HAZ. It would be inappropriate to interpret our finding as cause for dismissing improved sanitation as a potential pathway to improved nutrition more generally. For example, work based on 2006 and 2011 data from Nepal shows that the share of a district that is declared “Open Defecation Free” is associated with a small, but significant improvement in WHZ for children under five [57].

One caveat remains. Although these results are broadly consistent with recent work from Nepal and elsewhere, one must exercise caution when generalizing these findings beyond Nepal, given the specific geographical, economic, political and social conditions there. Furthermore, direct comparisons between our study and other research, even for Nepal, may be hindered by the fact that some research focuses on binary indicators of stunting and wasting (i.e. HAZ < − 2.0 and WHZ < − 2.0) as outcome variables, whereas we focus on the continuous z-scores.

## Conclusions

We set out in this paper to test two general hypotheses. In the first case, we find confirmatory evidence that the community-level factors we considered (food shortages, gender equity, proportion of population belonging to marginalized groups, commercialization of agriculture, distance to hospital and sanitation) are statistically relevant to explaining observed variance in height-for-age and weight-for-height, even when controlling for child- and household-level characteristics. Differences among districts are not as important as one might expect. However, that roughly one-fifth of *explained* variance occurs between districts for HAZ and more than one-third occurs between districts for WHZ provides evidence-based support for the idea that broad-based interventions targeting community-level factors could prove effective in promoting child growth in Nepal. No doubt, some of these factors reflect general improvements in local economic development, which over time contribute to the anthropometric patterns observed here. Furthermore, results from the three-level (child-household-district) model suggests that a nontrivial proportion of overall variance occurs between households, and would be misattributed to children in a child-district model. This suggests that some between-child variance in HAZ and WHZ occurs at a level that could be addressed by targeting households, and some occurs at a level that could be addressed by targeting communities. As a result, a 50-25-25 “rule of thumb” might be appropriate for policymakers in Nepal: when developing policies aimed at improving child growth outcomes, a starting point might be to focus 50% of efforts and attention directly on children, 25% on households, and 25% on communities, keeping in mind that the benefits of invested resources and the costs of those resources must be weighed, and might differ markedly across potential interventions.

Our second hypothesis was that omitting higher-level characteristics from models of height-for-age and weight-for-height would overestimate the importance of individual- and household-level factors (such as acute sickness, breastfeeding practices, mother’s education and health, and wealth) in explaining observed variance in growth metrics. We do not find strong statistical support for this view. Including community-level variables as district-level random slopes does not change lower-level parameter estimates very much, and interpretations remain remarkably stable. The results largely reinforce and underscore the importance of many traditional lower-level correlates with child growth. The main value of including these supra-household components is to account for a larger proportion of variance in outcomes that is unexplained in more parsimonious models.

This study raises several questions for further research, due largely to the ways in which multilevel models partition variance. Adding district-level covariates does not fully explain between-household variation, as the variance share at the household level remains fairly stable between specifications, representing approximately 20 to 30% of unexplained variation across all models. This suggests that including household-specific data on long-term food security and other omitted characteristics, which is not possible using DHS data alone, might improve the explanatory value of child growth models. Finally, this study cannot fully explain the statistically significant improvements in HAZ and WHZ observed in Nepal between 2006 and 2011. Understanding the source of these improvements probably requires more comprehensive longitudinal data than are currently available, although results from our multilevel framework reinforce existing understanding of the factors correlated with child growth. The flexibility of the multilevel approach appears to offer a wide array of benefits that make it a worthwhile tool for nutrition research based on observational data, especially in settings where the combination of child, household, and community features are of policy interest and concern.

## Additional file

Additional file 1: **Table S1.** Regression results for three-level (child-household-district) models of HAZ including birth year fixed effects **Table S2.** Regression results for three-level (child-household-district) models of WHZ including birth year fixed effects **Table S3.** OLS regression results for models of HAZ including birth year fixed effects **Table S4.** OLS regression results for models of WHZ including birth year fixed effects **Table S5.** Variance components for all variables, between districts and households. (DOCX 40 kb)
