# Supplementary material for: Multilevel analysis of individual, household, and community factors influencing child growth in Nepal
Source: BMC Pediatr. 2019 Apr 5;19:91. doi: 10.1186/s12887-019-1469-8 (PMC6449894; doi:10.1186/s12887-019-1469-8)
Supplement: Supplementary file 1 — Table S1. Regression results for three-level (child-household-district) models of HAZ including birth year fixed effects Table S2. Regression results for three-level (child-household-district) models of WHZ including birth year fixed effects Table S3. OLS regression results for models of HAZ including birth year fixed effects Table S4. OLS regression results for models of WHZ including birth year fixed effects Table S5. Variance components for all variables, between districts and households. (DOCX 40 kb) [file 12887_2019_1469_MOESM1_ESM.docx]

Supplemental Materials

Table S1 Regression results for three-level (child-household-district) models of HAZ including birth year fixed effects

Table S2 Regression results for three-level (child-household-district) models of WHZ including birth year fixed effects

Table S3 OLS regression results for models of HAZ including birth year fixed effects

Table S4 OLS regression results for models of WHZ including birth year fixed effects

Table S5 Variance components for all variables, between districts and households

Table S1 Regression results for three-level (child-household-district) models of HAZ, including birth year fixed effects

|  | Model 1 | Model 2 | Model 3 | Model 4 | Model 5 |
| --- | --- | --- | --- | --- | --- |
| Age  (months) | - | -0.621**  (0.221) | -0.535*  (0.231) | -0.578*  (0.228) | -0.530*  (0.229) |
| Age^2^  (months squared) | - | 0.152**  (0.0525) | 0.151**  (0.0524) | 0.153**  (0.0524) | 0.153**  (0.0524) |
| Female (0/1) | - | 0.00507  (0.0259) | 0.00660  (0.0259) | 0.00671  (0.0259) | 0.00649  (0.0259) |
| Twin (0/1) | - | -0.674***  (0.144) | -0.670***  (0.144) | -0.665***  (0.144) | -0.666***  (0.144) |
| Still breastfeeding (0/1) | - | 0.313  (0.253) | 0.321  (0.253) | 0.322  (0.253) | 0.322  (0.253) |
| Months breastfeeding  (months) | - | -0.202  (0.140) | -0.206  (0.140) | -0.206  (0.140) | -0.206  (0.140) |
| Months breastfeeding^2^  (months squared) | - | 0.0369  (0.0411) | 0.0381  (0.0411) | 0.0386  (0.0411) | 0.0385  (0.0411) |
| Fever in last two weeks (indicator) | - | 0.0222  (0.0350) | 0.0211  (0.0350) | 0.0215  (0.0350) | 0.0216  (0.0350) |
| Diarrhea in last two weeks (indicator) | - | -0.0702  (0.0406) | -0.0703  (0.0405) | -0.0703  (0.0405) | -0.0700  (0.0405) |
| Mother's education (years) | - | 0.162***  (0.0186) | 0.163***  (0.0186) | 0.163***  (0.0186) | 0.163***  (0.0186) |
| Access to handwashing (indicator) | - | 0.163***  (0.0339) | 0.158***  (0.0340) | 0.161***  (0.0339) | 0.161***  (0.0339) |
| Mother's BMI (BMI units) | - | 0.113***  (0.0153) | 0.112***  (0.0153) | 0.112***  (0.0153) | 0.113***  (0.0153) |
| Mother’s age at birth (years) | - | 0.00249  (0.0147) | 0.00233  (0.0147) | 0.00224  (0.0147) | 0.00214  (0.0147) |
| Wealth Index (quintile, 1-5, centered) | - | 0.0837***  (0.0153) | 0.0831***  (0.0153) | 0.0859***  (0.0153) | 0.0861***  (0.0153) |
| Water purification (0/1) | - | 0.0596  (0.0515) | 0.0559  (0.0514) | 0.0548  (0.0512) | 0.0571  (0.0512) |
| Year  (1=2011, 0=2006) | - | 0.866  (0.725) | 0.592  (0.760) | 0.731  (0.750) | 0.563  (0.753) |
| Altitude (m.a.s.l.) | - | -0.166***  (0.0268) | -0.179***  (0.0284) | -0.170***  (0.0265) | -0.175***  (0.0266) |
| Mother is a Dalit (0/1) | - | -0.123**  (0.0409) | -0.131**  (0.0411) | -0.121**  (0.0409) | -0.120**  (0.0409) |
| Biomass usage (0/1) | - | -0.0860  (0.0592) | -0.0895  (0.0593) | -0.0775  (0.0594) | -0.0813  (0.0595) |
| Constant | -1.913***  (0.0427) | -1.848**  (0.562) | -2.002***  (0.576) | -1.932***  (0.575) | -2.017***  (0.575) |
| Birth timing controls | Month, year | Month, year | Month, year | Month, year | Month, year |
| Residual variance | 1.367***  (.038) | 0.925***  (0.0268) | 0.925***  (0.0268) | 0.925***  (0.0268) | 0.925***  (0.0268) |
| District variance | 0.109***  (0.022) | 0.0325***  (0.0085) | 0.027**  (0.0086) | 0.001  (0.0073) | 1.15E-10  (5.72e-10) |
| Household Variance | 0.351***  (0.0360) | 0.401***  (0.0281) | 0.394***  (0.0281) | 0.394***  (0.0281) | 0.394***  (0.0281) |
| Food shortage† (% reporting shortage) | - | - | 0.0054*** (0.0049) | - | - |
| Gender equity† (female enrollment ratio) | - | - | 0.0088***  (0.0059) | 0.004***  (0.0054) | 0.0046***  (0.0056) |
| Marginal† (% marginalized) | - | - | - | 0.0236***  (0.0114) | 0.0234***  (0.01) |
| Commercial† (% selling food) | - | - | - | 0.00318*  (0.0079) | 0.00243  (0.0084) |
| Hospital distance† (minutes on foot) | - | - | - | 0.0120***  (0.0092) | 0.0118***  (0.0092) |
| ODF free†  (% of VDCs) | - | - | - | - | 0.00232***  (0.0034) |
| Observations | 7,533 | 7,533 | 7,533 | 7,533 | 7,533 |
| Total Variance | 1.827 | 1.3585 | 1.3464 | 1.312 | 1.319 |
| Level 1 R-squared | - | 0.3233 | 0.3233 | 0.3233 | 0.3233 |
| Level 3 R-squared | - | 0.7018 | 0.7486 | 0.9909 | 1 |
| Overall R-squared | - | 0.2564 | 0.2631 | 0.2775 | 0.2781 |
| District ICC | 0.0597 | 0.0239 | 0.0204 | 0.0007 | 8.75E-11 |
| Household ICC | 0.252 | 0.3191 | 0.313 | 0.2994 | 0.2986 |
| Log-Likelihood | -12743.5 | -11679.6 | -11674.3 | -11670.2 | -11669.5 |
| AIC | 25494.95 | 23449.25 | 23442.52 | 23448.44 | 23448.73 |

Note: Standard errors presented in parentheses. † indicates variable has been standardized.

*,**,***: Denotes statistical significance at the 10%, 5%, 1% confidence levels, respectively.

Use of these data did not require institutional review because respondents previously provided informed consent and were rendered anonymous before the data were released to us for analysis.

Table S2 Regression results for three-level (child-household-district) models of WHZ, including birth year fixed effects

|  | Model 1 | Model 2 | Model 3 | Model 4 | Model 5 |
| --- | --- | --- | --- | --- | --- |
| Age  (months) |  | -1.131***  (0.183) | -1.152***  (0.186) | -1.090***  (0.189) | -1.090***  (0.189) |
| Age^2^  (months squared) |  | 0.00502  (0.0463) | 0.00419  (0.0463) | 0.00185  (0.0463) | 0.00163  (0.0463) |
| Female (0/1) |  | 0.0104  (0.0228) | 0.0106  (0.0228) | 0.00942  (0.0228) | 0.00946  (0.0228) |
| Twin (0/1) |  | -0.464***  (0.124) | -0.467***  (0.124) | -0.464***  (0.124) | -0.465***  (0.124) |
| Still breastfeeding (0/1) |  | 0.228  (0.224) | 0.230  (0.223) | 0.235  (0.223) | 0.236  (0.223) |
| Months breastfeeding  (months) |  | -0.285*  (0.123) | -0.287*  (0.123) | -0.291*  (0.123) | -0.291*  (0.123) |
| Months breastfeeding^2^  (months squared) |  | 0.115**  (0.0362) | 0.115**  (0.0362) | 0.117**  (0.0362) | 0.117**  (0.0362) |
| Fever in last two weeks (0/1) |  | -0.134***  (0.0306) | -0.134***  (0.0306) | -0.135***  (0.0306) | -0.136***  (0.0306) |
| Diarrhea in last two weeks (0/1) |  | -0.113**  (0.0356) | -0.112**  (0.0356) | -0.112**  (0.0356) | -0.112**  (0.0356) |
| Mother's education (years) |  | 0.0416**  (0.0160) | 0.0405*  (0.0160) | 0.0408*  (0.0160) | 0.0409*  (0.0160) |
| Access to handwashing (indicator) |  | 0.0201  (0.0290) | 0.0190  (0.0291) | 0.0206  (0.0291) | 0.0206  (0.0291) |
| Mother's BMI (BMI units) |  | 0.231***  (0.0132) | 0.230***  (0.0132) | 0.231***  (0.0132) | 0.231***  (0.0132) |
| Mother’s age at birth (years) |  | -0.0216  (0.0127) | -0.0216  (0.0127) | -0.0209  (0.0127) | -0.0208  (0.0127) |
| Wealth Index (quintile, 1-5, centered) |  | 0.0130  (0.0130) | 0.0133  (0.0130) | 0.0132  (0.0130) | 0.0132  (0.0130) |
| Water purification (0/1) |  | 0.0725  (0.0440) | 0.0717  (0.0440) | 0.0734  (0.0440) | 0.0729  (0.0440) |
| Year  (1=2011, 0=2006) |  | 4.069***  (0.595) | 4.137***  (0.607) | 3.933***  (0.616) | 3.931***  (0.617) |
| Altitude (m.a.s.l.) |  | 0.161***  (0.0207) | 0.163***  (0.0213) | 0.156***  (0.0209) | 0.157***  (0.0208) |
| Mother is a Dalit (0/1) | - | -0.0491  (0.0349) | -0.0488  (0.0349) | -0.0469  (0.0349) | -0.0468  (0.0349) |
| Biomass usage (0/1) | - | 0.0651  (0.0505) | 0.0670  (0.0506) | 0.0669  (0.0505) | 0.0676  (0.0506) |
| Constant | -0.739***  (0.0316) | 1.518**  (0.474) | 1.559**  (0.479) | 1.440**  (0.483) | 1.437**  (0.483) |
| Birth timing controls | Month, year | Month, year | Month,  year | Month,  year | Month,  year |
| Residual variance | 0.85***  (0.0244) | 0.771***  (0.0223) | 0.771***  (0.0223) | 0.769***  (0.0222) | 0.769***  (0.0222) |
| District variance | 0.0574***  (0.0120) | 0.0137**  (0.00477) | 0.012**  (0.00481) | 0.0103  (0.00871) | 0.00929  (0.00907) |
| Household variance | 0.263***  (0.0240) | 0.231***  (0.0219) | 0.229***  (0.0219) | 0.23***  (0.0219) | 0.23***  (0.0219) |
| Food shortage† (% reporting shortage) | - | - | 0.00279***  (0.003) | - | - |
| Equity (% female in schools of total, standardized) | - | - | 0.0009**  (0.0021) | 0.0004  (0.0019) | 0.0004  (0.0019) |
| Marginal† (% marginalized) | - | - | - | 0.0014  (0.0075) | 0.002  (0.0077) |
| Commercial† (% selling food) | - | - | - | 1.41e-14***  (8.29e-14) | 1.40e-15***  (8.07e-15) |
| Hospital distance† (minutes on foot) | - | - | - | 0.0066***  (0.0048) | 0.0068***  (0.0049) |
| ODF free†  (% of VDCS) | - | - | - | - | 0.0005  (0.0019) |
| Observations | 7,533 | 7,533 | 7,533 | 7,533 | 7,533 |
| Total Variance | 1.1704 | 1.0157 | 1.012 | 1.0093 | 1.0083 |
| Level 1 R-squared |  | 0.0929 | 0.0929 | 0.0953 | 0.0953 |
| Level 3 R-squared |  | 0.7613 | 0.7909 | 0.8206 | 0.838153 |
| Overall R-squared |  | 0.1322 | 0.1353 | 0.1376 | 0.1385 |
| District ICC | 0.049 | 0.0135 | 0.0119 | 0.0103 | 0.0092 |
| Household ICC | 0.2740 | 0.2401 | 0.2386 | 0.2379 | 0.2371 |
| Log-Likelihood | -11086 | -10658.9 | -10658.1 | -10656.3 | -10656.2 |
| AIC | 22179.95 | 21407.88 | 21410.22 | 21469.43 | 21455.43 |

Note: Standard errors presented in parentheses. . † indicates variable has been standardized.

*,**,***: Denotes statistical significance at the 10%, 5%, 1% confidence levels, respectively.

Use of these data did not require institutional review because respondents previously provided informed consent and were rendered anonymous before the data were released to us for analysis.

Table S3 Regressions on HAZ with district clustered standard errors, including birth year fixed effects

|  | Model 1 | Model 2 | | Model 3 | Model 4 |
| --- | --- | --- | --- | --- | --- |
| Age  (months) | -0.785**  (0.23) | | -0.761**  (0.23) | -0.768**  (0.24) | -0.710**  (0.24) |
| Age^2^  (months squared) | 0.144*  (0.07) | | 0.139*  (0.07) | 0.141*  (0.07 | 0.141*  (0.07 |
| Female (0/1) | 0.011  (0.03) | | 0.011  (0.03) | 0.01  (0.03) | 0.01  (0.03) |
| Twin (0/1) | -0.649**  (0.21) | | -0.640**  (0.22) | -0.634**  (0.22) | -0.634**  (0.22) |
| Still breastfeeding (0/1) | 0.398  (0.27) | | 0.4  (0.27) | 0.39  (0.27) | 0.387  (0.27) |
| Months breastfeeding  (months) | -0.23  (0.14) | | -0.236  (0.15) | -0.231  (0.15) | -0.23  (0.15) |
| Months breastfeeding^2^  (months squared) | 0.042  (0.04) | | 0.044  (0.04) | 0.042  (0.04 | 0.042  (0.04 |
| Fever in last two weeks (0/1) | 0.038  (0.04) | | 0.043  (0.04) | 0.038  (0.04) | 0.036  (0.04) |
| Diarrhea in last two weeks (0/1) | -0.084*  (0.04) | | -0.085*  (0.04) | -0.082  (0.04) | -0.083  (0.04) |
| Mother's education (years) | 0.179***  (0.02) | | 0.167***  (0.02) | 0.168***  (0.02) | 0.168***  (0.02) |
| Access to handwashing (indicator) | 0.186***  (0.04) | | 0.175***  (0.04) | 0.173***  (0.04) | 0.173***  (0.04) |
| Mother's BMI (BMI units) | 0.112***  (0.01) | | 0.104***  (0.01) | 0.105***  (0.01 | 0.106***  -0.01 |
| Mother’s age at birth (years) | -0.009  (0.02) | | -0.011  (0.02) | -0.01  (0.02) | -0.01  (0.02) |
| Wealth Index (quintile, 1-5, centered) | 0.069**  (0.02) | | 0.074***  (0.02) | 0.073***  (0.02) | 0.074***  (0.02) |
| Water purification (0/1) | 0.082  (0.06) | | 0.07  (0.05) | 0.065  (0.05 | 0.067  (0.05 |
| Year  (1=2011, 0=2006) | 1.517  (0.79) | | 1.359  (0.8) | 1.351  (0.82) | 1.151  (0.82) |
| Altitude (m.a.s.l.) | -0.143***  (0.03) | | -0.156***  (0.03) | -0.167***  (0.03) | -0.170***  (0.03) |
| Mother is a Dalit (0/1) | -0.129*  (0.05) | | -0.133*  (0.05) | -0.133*  (0.05) | -0.130*  (0.05) |
| Biomass usage (0/1) | -0.095  (0.06) | | -0.112  (0.07) | -0.083  (0.06) | -0.088  (0.06) |
| Gender equity†  (Female enrollment ratio) |  | | 0.036  (0.03) | - | - |
|  | - | |  |  |  |
| Marginal† (% marginalized) | - | | 0.076**  (0.03) | 0.074**  (0.03 | 0.077**  (0.03) |
| Commercial† (% selling foodd) | - | | - | -0.03  (0.03) | -0.036  (0.03) |
| Hospital distance† (minutes on foot) | - | | - | 0.003  (0.03) | 0.001  (0.02) |
| ODF free †  (% of VDCs) | - | | - | 0.001  (0.04 | -0.002  (0.04) |
| Food shortage† (% reporting shortage, standardized) | - | | - | - | -0.027  (0.02) |
| Observations | 7,533 | | 7,533 | 7,533 | 7,533 |
| Constant | -1.568**  (0.57) | | -1.533*  (0.59) | -1.588**  (-0.59) | -1.704**  (-0.6) |
| $R^{2}$ | 0.256 | | 0.259 | 0.259 | 0.259 |

Note: Standard errors presented in parentheses. . † indicates variable has been standardized.

*,**,***: Denotes statistical significance at the 10%, 5%, 1% confidence levels, respectively.

Use of these data did not require institutional review because respondents previously provided informed consent and were rendered anonymous before the data were released to us for analysis.

Table S4 Regressions on WHZ with district clustered standard errors, including birth year fixed effects

|  | Model 1 | Model 2 | Model 3 | Model 4 |
| --- | --- | --- | --- | --- |
| Age  (months) | -1.039***  (0.21) | -1.066***  (0.2) | -0.973***  (0.2) | -0.951***  (0.2) |
| Age^2^  (months squared) | -0.003  (0.05) | -0.005  (0.05) | -0.007  (0.05) | -0.007  (0.05) |
| Female (0/1) | 0.009  (0.02) | 0.009  (0.02) | 0.007  (0.02) | 0.007  (0.02) |
| Twin (0/1) | -0.468***  (0.11) | -0.463***  (0.11) | -0.463***  (0.11) | -0.463***  (0.11) |
| Still breastfeeding (0/1) | 0.272  (0.25) | 0.27  (0.25) | 0.283  (0.25) | 0.282  (0.25) |
| Months breastfeeding  (months) | -0.297*  (0.14) | -0.299*  (0.14) | -0.305*  (0.14) | -0.305*  (0.14) |
| Months breastfeeding^2^  (months squared) | 0.115**  (0.04) | 0.116**  (0.04) | 0.117**  (0.04) | 0.117**  (0.04) |
| Fever in last two weeks (0/1) | -0.139***  (0.03) | -0.141***  (0.03 | -0.140***  (0.03 | -0.140***  (0.03 |
| Diarrhea in last two weeks (0/1) | -0.107**  (0.04) | -0.103**  (0.04) | -0.104**  (0.04) | -0.105**  (0.04) |
| Mother's education (years) | 0.045*  (0.02) | 0.038  (0.02) | 0.035  (0.02) | 0.035  (0.02) |
| Access to handwashing (indicator) | 0.01  (0.03) | -0.002  (0.03) | 0.005  (0.03) | 0.005  (0.03) |
| Mother's BMI (BMI units) | 0.245***  (0.02) | 0.238***  (0.02) | 0.238***  (0.02) | 0.239***  (0.02) |
| Mother’s age at birth (years) | -0.022  (0.01) | -0.023*  (0.01) | -0.023*  (0.01) | -0.023*  (0.01 |
| Wealth Index (quintile, 1-5, centered) | 0.009  (0.01) | 0.009  (0.01) | 0.009  (0.01) | 0.009  (0.01) |
| Water purification (0/1) | 0.104*  (0.05) | 0.091*  (0.04 | 0.089  (0.05 | 0.09  (0.05 |
| Year  (1=2011, 0=2006) | 3.800***  (0.72) | 3.804***  (0.69) | 3.589***  (0.68) | 3.512***  (0.66) |
| Altitude (m.a.s.l.) | 0.160***  (0.03) | 0.158***  (0.03) | 0.160***  (0.03) | 0.159***  (0.03) |
| Mother is a Dalit (0/1) | -0.062  (0.047) | -0.059  (0.047) | -0.064  (0.046) | -0.062  (0.047) |
| Biomass usage (0/1) | 0.044  (0.06) | 0.048  (0.06) | 0.046  (0.06) | 0.044  (0.06) |
| Food shortage† (% reporting shortage) | - | -0.024  (0.02) | - | - |
| Gender equity† (female enrollment ratio) | - | 0.054**  (0.02) | 0.062***  (0.02) | 0.063***  (0.02) |
| Marginal† (% marginalized) | - | - | -0.012  (0.02) | -0.015  (0.02) |
| Commercial† (% selling food) | - | - | 0.015  (0.02) | 0.015  (0.02) |
| Hospital distance† (minutes on foot) | - | - | -0.068  (0.03) | -0.069*  (0.03) |
| ODF free†  (% of VDCS) | - | - | - | -0.01  (0.02) |
| Observations | 7,533 | 7,533 | 7,533 | 7,533 |
| Constant | 1.364**  (0.41) | 1.421***  (0.4) | 1.211**  (0.43) | 1.166**  (0.42) |
| $R^{2}$ | 0.134 | 0.137 | 0.138 | 0.138 |

Note: Standard errors presented in parentheses. . † indicates variable has been standardized.

*,**,***: Denotes statistical significance at the 10%, 5%, 1% confidence levels, respectively.

Use of these data did not require institutional review because respondents previously provided informed consent and were rendered anonymous before the data were released to us for analysis.

Table S5 Variance components for all variables, between districts and households

|  | Household: | | District: | |
| --- | --- | --- | --- | --- |
| Variable | Between | Within | Between | Within |
| HAZ | 78.82% | 21.18% | 6.96% | 93.04% |
| WHZ | 79.77% | 20.23% | 6.01% | 93.99% |
| Age | 58.14% | 41.86% | 0.73% | 99.27% |
| Female | 71.15% | 28.85% | 1.03% | 98.97% |
| Breastfeeding | 54.73% | 45.27% | 1.22% | 98.78% |
| Breastfeeding duration | 80.62% | 19.38% | 1.94% | 98.06% |
| Fever in past two weeks | 78.49% | 21.51% | 2.72% | 97.28% |
| Diarrhea in past two weeks | 75.40% | 24.60% | 1.54% | 98.46% |
| Mother’s education | 97.59% | 2.41% | 14.73% | 85.27% |
| Hand washing access | 96.78% | 3.22% | 15.04% | 84.96% |
| Mother’s BMI | 95.76% | 4.24% | 10.96% | 89.04% |
| Mother’s age at birth | 91.96% | 8.04% | 3.99% | 96.01% |
| Wealth | 100.00% | 0.00% | 35.83% | 64.17% |
| Water purification | 100.00% | 0.00% | 17.92% | 82.08% |
| Altitude | 100.00% | 0.00% | 88.36% | 11.64% |
| Ethnicity (Dalit) | 99.90% | 0.10% | 9.90% | 90.10% |
| Biomass fuel use | 100.00% | 0.00% | 26.31% | 73.69% |

Use of these data did not require institutional review because respondents previously provided informed consent and were rendered anonymous before the data were released to us for analysis.
